# Pathogenesis and Risk Factors of Post-Infectious Bronchiolitis Obliterans in Children: A Focus on Adenovirus and Mycoplasma Infections

**DOI:** 10.3390/pathogens15050533

**Published:** 2026-05-14

**Authors:** Ling Zhu, Chenghao Mei, Chenchen Zhang, Jia Li, Daiyin Tian

**Affiliations:** 1 Department of Respiratory, Children’s Hospital of Chongqing Medical University, National Clinical Research Center for Child Health and Disorders, Ministry of Education Key Laboratory of Child Development and Disorders, Chongqing 400014, China; zhu_ling2025@163.com (L.Z.); linxmch@gmail.com (C.M.); chenchenz2026@163.com (C.Z.); jiaajiaalee@163.com (J.L.); 2 Chongqing Key Laboratory of Child Rare Diseases in Infection and Immunity, Chongqing 400014, China; 3 Department of Respiratory, Children’s Hospital of Fudan University, Shanghai 201100, China

**Keywords:** post-infectious bronchiolitis obliterans, children, inflammation, risk factor, pathogenic mechanisms

## Abstract

Post-infectious bronchiolitis obliterans (PIBO) is a severe chronic airway disease in children following lower respiratory tract infections. Human adenovirus (HAdV) and Mycoplasma pneumoniae (MP) are the major associated pathogens, with geographic variations in their relative importance. This review analytically compares the mechanistic divergence and convergence between HAdV and MP. Both pathogens converge on MyD88/NF-κB/MAPK signaling and neutrophil-driven inflammation, but diverge in initial host engagement (CAR/integrins vs. TLR2/6 and CARDS toxin) and inflammasome activation (TLR9-related vs. NLRP3-related). This review aims to propose an integrative model linking acute immune activation to fibrotic bronchiolar narrowing and to evaluate the risk factors for PIBO. Genetic susceptibility and epigenetic regulation help explain population differences in PIBO risk and geographic distribution. Despite progress, significant knowledge gaps remain, including the lack of single-cell resolution studies, the absence of co-infection animal models, and uncertainty regarding the long-term efficacy of targeted immunomodulatory therapies. Addressing these gaps is essential for improving early diagnosis and clinical outcomes.

## 1. Introduction

Bronchiolitis obliterans (BO) is a rare, chronic lung condition caused by bronchiolar epithelium injury, leading to inflammation, progressive small airway fibrosis, and narrowing or even complete obstruction [1,2]. This pathological process originates from injury to the lower respiratory tract. BO appears in various contexts, including post-transplant complications (lung, heart-lung, bone marrow), autoimmune disorders (like rheumatoid arthritis, Sjogren’s syndrome), exposure to toxic fumes, airway pathogens, and drug reactions. In the pediatric population, infections are the predominant trigger for BO, with post-infectious bronchiolitis obliterans (PIBO) being the most common form observed in children. PIBO typically develops after a severe lower respiratory tract infection in previously well children. Among the reported infectious triggers, human adenovirus (HAdV) has been one of the most consistently associated pathogens, particularly in pediatric cohorts from South America and East Asia [3,4,5]. Mycoplasma pneumoniae (MP) is increasingly recognized as an additional major PIBO-associated pathogen, with particularly strong evidence from East Asian cohorts and growing support from other regions [6,7]. The prevalence of the disease is unknown, but PIBO is more common in children than adults [8]. Reported PIBO cases are concentrated in East Asian and South American cohorts, but whether this pattern reflects biological susceptibility, regional pathogen epidemiology, referral bias, or differences in diagnostic recognition remains uncertain [4,7,8,9]. Current understanding of the mechanisms responsible for PIBO remains limited. In addition, no unified standardized protocol exists for PIBO management, and high-quality clinical evidence to support therapeutic regimens is insufficient. The insidious onset, delayed diagnosis, and absence of effective targeted therapies collectively contribute to the generally unfavorable prognosis in affected children. Children with PIBO commonly suffer from recurrent wheezing, secondary pneumonia episodes, recurrent atelectasis, obstructive exacerbations, frequent hospitalizations, and, in severe cases, even death.

Currently, the literature lacks a comprehensive overview of PIBO progression trajectories, and few studies have systematically summarized existing research on the pathogenesis of childhood PIBO [1,4,10]. Nonetheless, exploring its underlying immunological mechanisms and relevant risk factors is essential for developing targeted prevention measures and implementing early clinical interventions. Therefore, this review aims to elucidate the pathogenesis and risk factors associated with childhood PIBO, to link acute infectious injury to persistent airway remodeling, and to systematically compare pathogen-specific immune responses.

This narrative review was informed by literature searches in PubMed, Web of Science, and Embase up to April 2026 using combinations of the terms ‘post-infectious bronchiolitis obliterans’, ‘bronchiolitis obliterans’, ‘children’, ‘adenovirus’, ‘HAdV’, ‘Mycoplasma pneumoniae’, ‘airway remodeling’, ‘fibrosis’, and ‘risk factors‘. Priority was given to pediatric cohorts, mechanistic studies, systematic reviews, and clinically informative recent updates.

## 2. Pathogen-Specific Inflammation in PIBO

**Figure 1:**
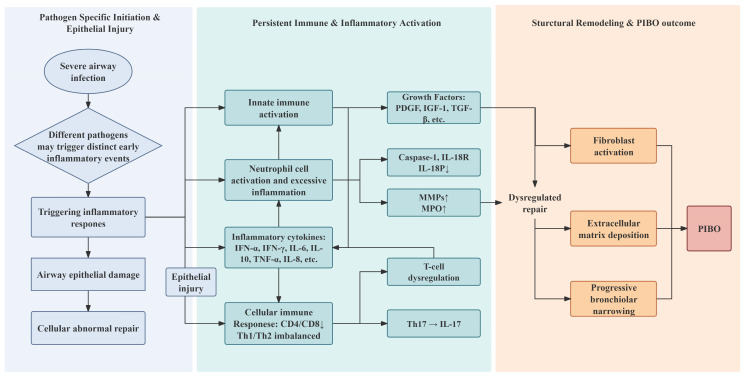
Progression stages of PIBO from initial insult to structural remodeling. The sequence begins with severe airway infection, where different pathogens may trigger distinct early inflammatory events, leading to airway epithelial damage and abnormal cellular repair. The secretion of pro-inflammatory cytokines and chemokines sustains a state of persistent inflammation, which eventually leads to significant airway remodeling and fibrosis. ↑ indicates increase; ↓ indicates decrease. PIBO, post-infectious bronchiolitis obliterans; IFN, interferon; IL, interleukin; TNF, tumor necrosis factor; PDGF, platelet-derived growth factor; IGF-1, insulin-like growth factor-1; TGF-β, transforming growth factor-β; IL-18R, interleukin-18 receptor; IL-18BP, interleukin-18 binding protein; MMPs, matrix metalloproteinases; MPO, myeloperoxidase.

Pathogen-specific inflammatory signaling is central to PIBO pathobiology, but as shown in Figure 1, its clinical relevance lies in whether acute immune activation progresses to persistent epithelial injury, dysregulated repair, fibroblast activation, and fixed bronchiolar narrowing. The exact mechanisms may vary depending on the etiological agent, as different pathogens trigger distinct immune responses. This variability explains the differences in diagnostic and prognostic biomarkers, as well as the risk factors for PIBO, depending on the causative agents [11]. HAdV infection remains one of the most strongly associated infectious antecedents of childhood PIBO, particularly after severe pneumonia requiring intensive respiratory support [4,12,13]. The invasion, replication, and release of HAdV represent a complex process. Other respiratory viruses, including respiratory syncytial virus (RSV) and influenza virus, have been sporadically reported as causes of PIBO in certain geographic regions, though at considerably lower frequencies than HAdV [14,15]. The relative contribution of these pathogens to the overall PIBO burden, particularly in settings where HAdV circulation is low, remains poorly characterized and warrants further epidemiological investigation.

### 2.1. HAdV-Induced Inflammation

HAdV initiates infection by binding to cell surface receptors, primarily the coxsackievirus and adenovirus receptor (CAR), which is crucial for cell–cell interactions. Certain HAdV types, such as HAdV-3, HAdV-7, and HAdV-11, can also interact with CD80 or CD86 [16,17]. This binding is facilitated by Arginine-Glycine-Aspartic acid (RGD) peptides on the viral penton base, which interact with cellular integrins, promoting cellular adhesion and endocytosis [18,19]. Once inside the cell, the virus is transported to the nucleus, where it hijacks the host cell machinery for replication and assembly [20,21]. The release of HAdV triggers downstream signaling pathways, leading to the production of inflammatory cytokines and chemokines that cause significant lung tissue damage.

**Figure 2:**
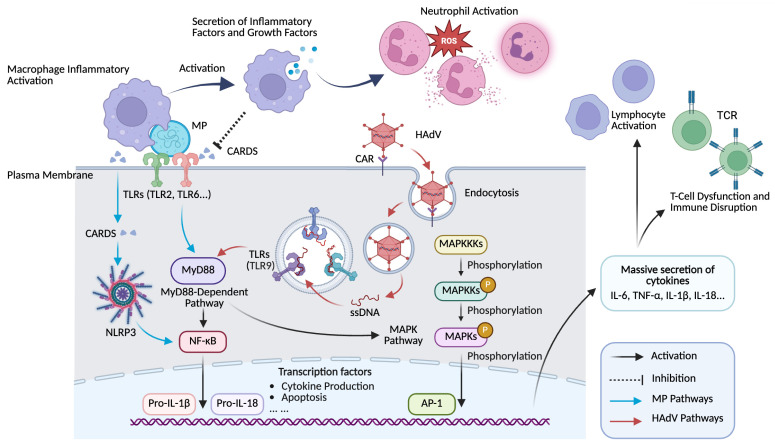
Mechanistic convergence and divergence of HAdV and MP in host cell engagement. HAdV utilizes the CAR for attachment and enters the host cell through endocytosis, where it subsequently interacts with TLR9 to initiate intracellular signaling. In contrast, MP engages host cells via TLR2/6 and the CARDS toxin, with the latter activating the NLRP3 inflammasome to trigger the inflammatory response. These distinct triggers converge on shared signaling pathways including MyD88-dependent signaling, NF-κB, and MAPK to drive the massive release of cytokines and chemokines. The resulting molecular cascade sustains the cellular immune disorders that characterize the disease. … indicates additional downstream effects not listed. MP, Mycoplasma pneumoniae; HAdV, human adenovirus; TLR, Toll-like receptor; CARDS, Community-Acquired Respiratory Distress Syndrome; MyD88, myeloid differentiation factor 88; MAPK, mitogen-activated protein kinase; MAPKKK, MAPK kinase kinase; MAPKK, MAPK kinase; NF-κB, nuclear factor-kappa B; AP-1, activator protein 1; IL, interleukin; TNF, tumor necrosis factor; ROS, reactive oxygen species.

Previous research has indicated that Toll-like receptor 9 (TLR-9) is the predominant Toll-like receptor for HAdV [22]. Tang Z et al. revealed that high mobility group box-1 (HMGB1), an inflammatory mediator released by necrotic cells, promotes HAdV-7 replication and plays a crucial role in HAdV infection [23]. Furthermore, HMGB1 interacts with multiple membrane receptors, including the receptor for advanced glycation end-products (RAGE), TLR-2, TLR-4, and TLR-9 [22,23,24,25,26,27]. This interaction initiates the myeloid differentiation factor 88 (MyD88)-dependent signaling pathway, which subsequently triggers two distinct downstream cascades. The first is the mitogen-activated protein kinase (MAPK) pathway. The second is the nuclear factor-kappa B (NF-κB) pathway [27,28]. Consequently, the expression of pro-inflammatory cytokines is upregulated, setting off a cascade of immune and inflammatory responses. High levels of these cytokines and chemokines can induce cell damage and mediate destructive tissue inflammation [29,30]. Chen Q et al. observed a significant increase in multiple cytokines in lung tissues infected with HAdV-7, including inducible protein-10 (IP-10), macrophage inflammatory protein 1β (MIP-1β), monokine induced by gamma interferon (MIG), MIP-1α, monocyte chemoattractant protein-1 (MCP-1), interferon-α (IFN-α), IFN-γ, IL-6, IL-10, and TNF-α. Notably, HAdV-7 induced higher levels of IL-10, MIP-1α, and MIP-1β than did HAdV-3 [31]. Collectively, these findings suggest that severe HAdV infection may establish a pro-fibrotic repair milieu through the convergence of extensive epithelial injury, high viral burden, and a cytokine profile skewed toward monocyte/macrophage-driven responses, thereby increasing the risk of aberrant bronchiolar remodeling [12,23,31,32,33]. Additionally, Xie L et al. found that HAdV-7 had a higher average viral load than other serotypes (except HAdV-3), which may contribute to the observed variations in disease severity [33]. The HAdV-specific entry and TLR9 signaling pathways are depicted in Figure 2.

### 2.2. MP-Induced Inflammation

Mycoplasma pneumoniae (MP) is a significant pathogen in pediatric community-acquired pneumonia (CAP) and an important contributor to PIBO, particularly in East Asia. The role of MP in PIBO has gained increasing recognition, with studies from Malaysia and Korea identifying it as the second most common etiology after HAdV infection [34,35,36]. Severe or refractory cases of MP pneumonia, especially those unresponsive to macrolides, are prevalent in East Asia and can lead to atelectasis, bronchiectasis, and PIBO [34,35,36]. Like HAdV, MP may persist within airway epithelial cells, potentially sustaining low-grade inflammation and contributing to chronic airway remodeling. Recognition of MP as a PIBO-associated pathogen has expanded in recent years, but the literature remains geographically imbalanced.

The intrapulmonary pathogenesis of MP is complex. During the initial phase, MP adheres to the bronchial epithelium via its terminal structure. This adhesion induces intracellular metabolic and ultrastructural changes in infected cells, disrupts cytoskeletal organization, and depletes host cell nutrients, ultimately resulting in ciliary stasis and cell death [37]. This adhesion process is multifactorial, involving surface proteins such as P1, P30, P90, P40, and P116 [38,39,40,41]. MP also invades host cells, releases reactive oxygen species that cause direct cellular damage, and induces cytokine production, resulting in inflammatory injury [37].

The Community-Acquired Respiratory Distress Syndrome (CARDS) toxin of MP is a key virulence factor that activates inflammasomes and plays a crucial role in MP-induced lung injury [42,43,44,45]. CARDS toxin binds to the surfactant protein A (SP-A) receptor and is internalized, leading to vacuolar degeneration of airway epithelial cells, impaired ciliary motility, and loss of epithelial integrity [45,46]. The toxin promotes inflammasome activation via ribosylation, which triggers the NLRP3 inflammasome and facilitates the maturation and release of pro-inflammatory cytokines, including IL-1β and IL-18 [47]. Additionally, CARDS toxin-induced inflammasome activation may be relevant to PIBO because repeated epithelial injury, amplified IL-1 family signaling, and downstream non-resolving inflammation can create a microenvironment that favors aberrant repair and airway fibrosis [48]. IL-1β, in turn, activates NF-κB-dependent pathways, amplifying the inflammatory response [40,49,50]. CARDS toxin also induces the secretion of T-helper 2 (Th2)-associated cytokines such as interleukin-3 (IL-3), IL-4, and TNF-α, thereby enhancing the Th2-mediated immune response in children with MP pneumonia (MPP) [51,52,53].

MP components, including lipids and lipoproteins, act as ligands for TLR4 and TLR2/6. They stimulate macrophage autophagy and activate both the NLRP3 inflammasome and the NF-κB pathway [37,54,55]. Lipoproteins are primarily recognized by TLR2, together with TLR6 or TLR1, via their leucine-rich repeat (LRR) regions. This recognition activates MyD88-dependent signaling, leading to the activation of NF-κB, MAPKs, and activator protein 1 (AP-1). Through mechanisms similar to those described for HAdV, these MP components induce immune cells to produce a range of pro-inflammatory cytokines [56,57,58,59,60]. Figure 2 dissects the mechanistic convergence and divergence between HAdV and MP at the molecular and cellular levels.

### 2.3. Cellular Immune Disruptions and T Lymphocyte Dynamics

Several studies have reported cellular immune abnormalities in children with PIBO [61,62,63,64]. Research from Brazil demonstrated that both B and T lymphocytes are involved in the pathogenesis of childhood PIBO, with T cells being the predominant infiltrating cell type in the airways [61]. Among these, CD3^+^ T cells are the most frequently observed, and the CD8^+^ T cell subset predominates. Cazzato S et al. documented elevated levels of peripheral blood CD8^+^ T cells, HLA-DR+ CD8+ T cells, and serum interferon-gamma (IFN-γ) in children with HAdV infection, suggesting that activated CD8^+^ T cells may serve as an immunological marker of disease severity [62].

CD8^+^ T cells can contribute to tissue damage through multiple cytotoxic mechanisms. They release granule enzymes such as granzyme B and perforin, which induce apoptosis of target cells, including airway epithelial cells [61]. They also secrete chemokines that recruit additional inflammatory cells, thereby amplifying local inflammation. Furthermore, activated CD8^+^ T cells produce IFN-γ, which can sustain macrophage activation and further promote the release of pro-fibrotic mediators [62,63,64,65]. These combined actions may perpetuate epithelial injury and create a microenvironment conducive to chronic airway remodeling.

Consistent with these observations, subsequent work by Cazzato S et al. found that PIBO patients have a decreased CD4/CD8 T-cell ratio [63]. Given that CD4^+^ T cells comprise both Th1 and Th2 subsets, alterations in the CD4/CD8 ratio are accompanied by shifts in the Th1/Th2 balance. Although Th1/Th2 imbalance has been implicated in other forms of BO, studies indicate that in pediatric PIBO, peripheral blood levels of Th1-associated IFN-γ and Th2-associated IL-4 and IL-10 do not differ significantly from healthy controls, suggesting that Th1/Th2 dysregulation may not be a major driver in this specific context [48]. Taken together, these findings suggest that PIBO involves a persistent adaptive immune imbalance characterized by cytotoxic-leaning T-cell responses, reduced CD4/CD8 ratios, and sustained chemokine amplification, all of which may reinforce epithelial injury and chronic airway remodeling.

### 2.4. Neutrophil-Driven Inflammation

**Figure 3:**
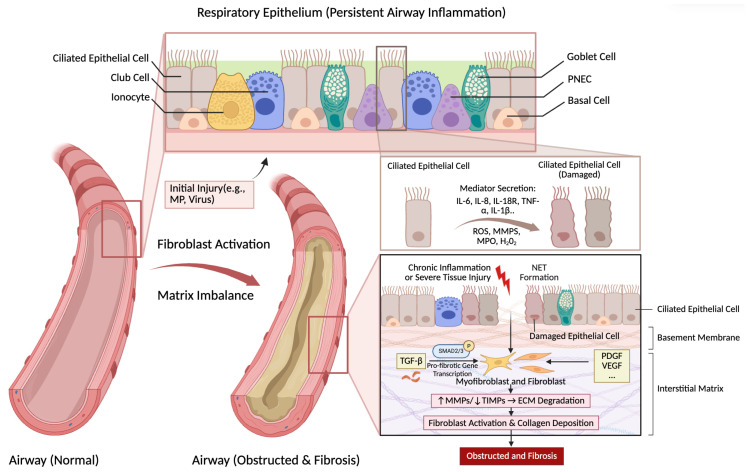
Persistent airway inflammation, fibroblast activation, and matrix imbalance in PIBO. Initial injury (e.g., by MP or virus) damages the respiratory epithelium. Damaged epithelial cells secrete mediators such as IL-6, IL-8, IL-18R, TNF-α, and IL-1β, as well as ROS, MMPs, MPO, and H_2_O_2_. NETs also contribute to epithelial injury. Chronic inflammation or severe tissue injury triggers the release of growth factors (TGF-β, PDGF, VEGF) and disruption of the MMP/TIMP balance. Specifically, TGF-β promotes SMAD2/3 phosphorylation and pro-fibrotic gene transcription. An increased MMP/TIMP ratio triggers ECM degradation, further driving fibroblast activation and collagen deposition. These processes result in the characteristic airway obstruction and fibrosis. ↑ indicates increase; ↓ indicates decrease; … indicates additional factors not listed. PIBO, post-infectious bronchiolitis obliterans; MP, Mycoplasma pneumoniae; IL, interleukin; IL-18R, interleukin-18 receptor; TNF-α, tumor necrosis factor-α; ROS, reactive oxygen species; MMPs, matrix metalloproteinases; MPO, myeloperoxidase; TGF-β, transforming growth factor-β; PDGF, platelet-derived growth factor; VEGF, vascular endothelial growth factor; TIMP, tissue inhibitor of metalloproteinases; ECM, extracellular matrix; PNEC, pulmonary neuroendocrine cell; NETs, neutrophil extracellular traps.

Neutrophils are the dominant effectors of small airway inflammation in PIBO [66], with interleukin-8 (IL-8) acting as the pivotal orchestrator of their recruitment and activation. Clinical data consistently show a robust correlation between elevated IL-8 levels in induced sputum and heightened neutrophil counts [67,68,69,70], emphasizing IL-8 as a central driver of pathogenesis. As shown in Figure 3, once activated, neutrophils engage in a respiratory burst that generates reactive oxygen species (ROS), inducing severe oxidative stress and damaging the respiratory epithelium. Simultaneously, degranulation releases proteolytic enzymes, such as matrix metalloproteinases (MMPs) and neutrophil elastase, which degrade the lung parenchyma and perpetuate the inflammatory cycle [66,71]. Antineutrophil cytoplasmic antibodies (ANCA) can also activate neutrophils, triggering a respiratory burst and promoting the production of pro-inflammatory cytokines (including IL-6 and IL-8) as well as endothelial damage [72,73,74].

**Figure 4:**
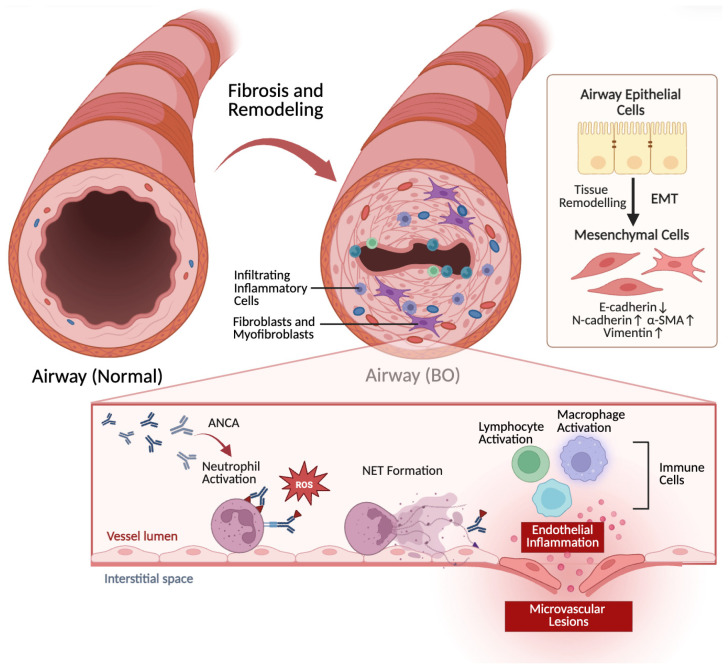
Airway remodeling and microvascular lesions in bronchiolitis obliterans. A normal bronchiole is shown on the left, with intact airway epithelial cells, normal interstitial space and vessel lumen. In PIBO (right), inflammatory infiltration (macrophage activation, lymphocyte activation, neutrophil activation, and NET formation) drives EMT, characterized by the downregulation of E-cadherin and upregulation of N-cadherin, α-SMA, and vimentin, converting epithelial cells into mesenchymal cells. Fibroblasts and myofibroblasts proliferate, leading to tissue remodeling. Microvascular lesions include endothelial inflammation, immune cell accumulation in the interstitial space, and vessel lumen changes. These combined processes result in the characteristic fibrotic occlusion of the small airway. ↑ indicates upregulation; ↓ indicates downregulation. PIBO, post-infectious bronchiolitis obliterans; NET, neutrophil extracellular trap; EMT, epithelial–mesenchymal transition; α-SMA, α-smooth muscle actin.

Recent transcriptomic insights highlight the role of neutrophil extracellular traps (NETs) in activating epithelial–mesenchymal transition (EMT) pathways in small airway epithelial cells (SAECs). This finding suggests that neutrophils directly facilitate the transition from acute inflammation to structural airway remodeling [75]. The failure of pro-resolving pathways to clear these inflammatory cells allows the acute response to evolve into the chronic obstructive lesions characteristic of PIBO. The role of NETs in this transition is summarized in Figure 4, where neutrophil activation and NET formation are linked to microvascular lesions and airway obstruction.

### 2.5. Macrophage-Mediated Inflammation and Fibrosis

Macrophages serve as critical intermediaries between innate and adaptive immunity in the pathogenesis of BO. They are recruited in response to neutrophil chemotaxis and, once activated, secrete a variety of inflammatory mediators that amplify neutrophil chemotaxis, aggregation, and adhesion. Activated macrophages also release growth factors such as platelet-derived growth factor (PDGF), insulin-like growth factor-1 (IGF-1), and transforming growth factor-β (TGF-β), thereby actively participating in fibrosis and airway remodeling [66].

### 2.6. Failure of Inflammatory Resolution and Th17/Treg Imbalance

The failure of inflammatory resolution is a critical turning point in the progression from acute inflammation to chronic PIBO. The imbalance between Th17 and regulatory T cells (Tregs) is involved in the development of airway fibrosis. Th17 cells drive autoimmune responses and inflammatory reactions, whereas Tregs restrain inflammation and preserve immune homeostasis [48]. In the airway microenvironment of PIBO, this disturbed balance hinders the resolution of inflammation and fosters fibrotic progression.

Th17 cells and their cytokine IL-17 have been implicated in bronchiolitis obliterans syndrome (BOS) after lung transplantation [76]. In children with PIBO, the proportion of Th17 cells in bronchoalveolar lavage fluid is significantly increased and positively correlates with declining lung function, suggesting overlapping mechanisms between PIBO and post-transplant BOS [77]. IL-17 promotes neutrophil recruitment and type V collagen production. Together with IL-18, it can upregulate IL-6 and IL-8, thereby activating the NF-κB pathway [78,79,80].

Recent research highlights that specialized pro-resolving mediators (SPMs) including resolvin D1, resolvin D2, and maresin 1 are critical for actively regulating the Th17/Treg balance [81,82,83]. These mediators reduce Th17 differentiation by downregulating transcription factors such as Rorc and simultaneously promote the generation of Foxp3+ Tregs [81]. The role of Tregs is complex and context-dependent. Tregs can suppress inflammation by secreting IL-10 and TGF-β [84,85,86], and may protect against airway fibrosis by facilitating epithelial repair and inhibiting proinflammatory mediators. They can also promote fibrosis by secreting PDGF and TGF-β, which enhance epithelial–mesenchymal transition (EMT) and fibroblast activation [48].

These insights suggest that therapies aimed at restoring Th17/Treg balance or enhancing proresolving pathways, for example, using IL-10 or Treg-enhancing agents, hold promise for PIBO [84,85].

## 3. Fibroblast Activation, Matrix Remodeling, and Epithelial–Mesenchymal Transition

### 3.1. Fibroblast Activation and Matrix Imbalance

Fibroblasts are essential for tissue fibrotic repair. Inflammation and infection damage airway epithelial cells, leading to depletion of airway progenitor cells and the initiation of aberrant repair responses. Fibrous and purulent exudates then accumulate in the airway lumen, further triggering myofibroblasts to deposit collagen and mucopolysaccharides into the extracellular matrix. An imbalance between matrix metalloproteinases (MMPs) and their inhibitors (TIMPs) contributes to fibrosis and airway remodeling in PIBO. Excessive MMP expression, mainly secreted from activated neutrophils, degrades the extracellular matrix (ECM), promoting fibroblast infiltration, proliferation, and activation, as well as the release of VEGF and TGF-β. This process drives airway fibrosis [87] (Figure 3, lower part).

### 3.2. Epithelial–Mesenchymal Transition and Molecular Markers

Epithelial–mesenchymal transition (EMT) represents a pivotal phenotypic reprogramming in PIBO, where stationary epithelial cells lose their polarity and transform into invasive, contractile myofibroblasts. This process is characterized by the downregulation of epithelial markers, such as E-cadherin, and the concomitant upregulation of mesenchymal markers including N-cadherin, α-smooth muscle actin (α-SMA), and Vimentin [88].

### 3.3. Fibroblast Activation and Molecular Pathways

The central driver of fibrotic remodeling in PIBO is the persistent activation of fibroblasts, primarily orchestrated by the TGF-β/SMAD signaling pathway. Upon activation by tissue injury or inflammatory stimuli, TGF-β1 binds to its receptors (TβRI/II), leading to the phosphorylation of SMAD2 and SMAD3. These phosphorylated SMADs form a heteromeric complex with SMAD4, which translocates into the nucleus to initiate the transcription of pro-fibrotic genes, including collagen types I and III [88]. Beyond the classical SMAD pathway, fibroblast activation in PIBO is intensified through extensive crosstalk with inflammatory cascades [89].

Recent studies have identified epigenetic regulation as an additional critical layer in maintaining the fibrotic phenotype. Epigenetic modifications, including DNA hypermethylation of anti-fibrotic genes and the dysregulation of microRNAs (miRNAs), contribute to the irreversible nature of fibroblast-to-myofibroblast differentiation. For example, reduced expression of miR-29 family members has been linked to the failure of ECM degradation control. Additionally, histone acetylation status modulated by p300/CBP hub enzymes integrates metabolic signals with fibrotic gene expression. This multi-layered regulatory network, involving SMAD-dependent signaling, inflammatory interactions, and epigenetic shifts, underpins the robust cellular memory of fibrotic cells in PIBO, explaining why standard anti-inflammatory therapies often fail to reverse established airway obliteration [90].

## 4. Microvascular Alterations

The microvascular lesions that occur during BO are mainly caused by abnormal vascular regeneration and remodeling mediated by immune and inflammatory responses. As shown in Figure 4, immune and inflammatory reactions lead to microvascular endothelial ischemic and hypoxic injury, endothelial inflammation, abnormal microvascular regeneration, fibrosis of terminal capillaries, and microvascular remodeling. The primary manifestation is ischemic injury to vascular endothelial cells, encompassing endothelial inflammation and fibrotic constriction of small terminal vessels, both of which impair small airway function. An increase in inflammatory mediators such as TGF-β and VEGF can promote the occurrence of airway fibrosis and occlusive lesions [80,91].

## 5. Persistent Airway Inflammation and Fibrosis

These pathogens, particularly MP and HAdV, can persist within the host for an extended period, with ongoing airway inflammation leading to continuous formation of peribronchial scars and fibrosis. Figure 3 illustrates the cellular events underlying persistent airway inflammation and the transition to fibrosis, while Figure 4 contrasts a normal bronchiole with the characteristic obstructive and fibrotic changes seen in PIBO. Studies have demonstrated that the development of BO is associated with an increase in fibroblast-like cells expressing type III procollagen mRNA. As the occlusion of the lumen progresses, these cells become dominant. These fibroblasts become excessively activated, secreting a substantial amount of collagen and extracellular matrix, which deposits on the walls of the small bronchi. With the ongoing progression of airway inflammation, fibrosis around the small airways and intraluminal scarring continue to form, leading to centripetal narrowing and destruction of the bronchiole lumens. Necrotic and desquamated epithelial cells, inflammatory exudates, and fibrotic debris may obstruct bronchi, bronchioles, or alveolar ducts following organization, leading to irreversible airflow limitation and ultimately the pathological picture of BO [61,92].

## 6. Risk Factors for PIBO

Current research on risk factors for PIBO is limited. A major challenge is that predictive variables, severity markers, treatment exposures, and causal determinants are often grouped together under the broad label of risk factors. Predictive model variables help make accurate predictions, but do not necessarily cause the outcome. In contrast, true risk factors require evidence of a causal relationship [93]. Variables such as prolonged fever, hypoxemia, long hospitalization, pleural effusion, extensive consolidation, and atelectasis are better interpreted primarily as markers of severe acute airway injury unless supported by stronger causal analyses [6,11,34,94] (Table 1). Notably, while hypoxemia shows the strongest correlation with PIBO development, it remains to be explored whether it serves as a direct driver of pathogenesis or merely a surrogate for severe disease [11,94]. Elevated lactate dehydrogenase (LDH), mechanical ventilation, and use of corticosteroids or γ-globulins have also been reported as potential risk factors. These indicators likely represent the intensity of the acute lung injury, though they may also contribute to PIBO development through specific pathogenic pathways. However, the reported associations must be interpreted with caution. For instance, the link between mechanical ventilation and PIBO often reflects confounding by indication, as ventilatory support is typically reserved for children with the most severe acute disease [94]. Similarly, the role of corticosteroids and γ-globulins remains obscured by similar biases in retrospective cohorts. Although corticosteroids are commonly used for their anti-inflammatory properties, their safety and efficacy have not been established in controlled trials [95]. Consequently, further high-quality research into the optimal timing, duration, and dosage of these therapies is essential to provide a robust basis for clinical treatment. Recent literature suggests that atopic conditions may be a risk factor for PIBO, and the co-existence of PIBO and asthma in some patients points to a potential link between these conditions, possibly mediated by shared inflammatory pathways or genetic predispositions [96].

Mechanistically, co-infection with multiple pathogens (e.g., HAdV, MP, and other pathogens) may act as a credible risk factor by activating broader immune pathways. HAdV infection, in particular, is a well-established driver of persistent airway damage. HAdV can sustain an inflammatory state through the production of early viral proteins like E1A and non-coding RNAs, which amplify host inflammatory genes. Furthermore, HAdV binds to the cellular protein FUBP1 to suppress the p53 stress response, thereby inhibiting the host’s normal antiviral immune response [97]. This allows for persistent infectious damage even in the absence of active viral replication [61,97]. Hogg [98] suggested that latent HAdV infection expressing E1A could stimulate connective-tissue growth and transform acute bronchiolitis into PIBO by activating inflammation-amplifying genes. Viruses may also trigger autoimmune-like reactions through molecular mimicry, lymphocyte activation, or exposure of host self-epitopes [61].

Genetic susceptibility and epigenetic regulation are crucial emerging factors in explaining PIBO risk and its geographic distribution [99,100]. Variants in the mannose-binding lectin 2 (MBL2) gene, such as the rs1800450 B-allele, lead to low circulating MBL protein levels and impaired innate immunity [101,102,103,104]. High frequencies of this B-allele in certain South American populations correlate with the historically higher incidence of PIBO in countries like Argentina and Chile [4,105,106]. Alongside MBL2 deficiency, genetic variants in DNAH9 and aberrant miRNA expression profiles are also associated with PIBO pathogenesis. DNAH9 mutations impair ciliary motility and mucin clearance while altering cytokine profiles (e.g., reducing IL-1α and TNF-α), which facilitates persistent lung lesions [107]. Simultaneously, the downregulation of specific miRNAs, such as miR-335-5p and miR-186-5p, exacerbates inflammatory and fibrotic processes by disinhibiting the TGF-β and FoxO signaling pathways, thereby driving irreversible airway remodeling [100,108,109]. These genetic and epigenetic insights provide a more precise framework for understanding population-level differences in PIBO incidence without relying on imprecise racial categories.

**Table 1:** Risk Factors and Prediction Models for PIBO in children.

Study Author, Year	Country	Pathogens	Sample Size (PIBO/Total)	Multiple-Factor Analysis	AUC
Xu, W. et al. (2024) [96]	China	HAdV	66/131	Co-infection, atopic conditions, and duration of fever	NA
Wen S et al. (2024) [110]	China	HAdV	36/148	Aged <1 year, admission to PICU, long duration of fever, and bilateral lung infection	0.85 (95% CI, 0.78–0.92)
Yan S et al. (2023) [111]	China	Unlimited	78/228	Age of patients, length of hospital stay, mechanical ventilation, HAdV, and IL-2 level	0.907 (95% CI, 0.888–0.926)
Peng L et al. (2023) [112]	China	HAdV	46/863	Univariate analysis only: duration of fever, invasive mechanical ventilation, complications, and N%; Male, duration of fever, HAdV load, and fungi coinfection (HAdV cases with IMV)	0.857 (95% CI, 0.744–0.928)
Yuan J et al. (2023) [113]	China	HAdV	28/112	Respiratory support, length of wheezing days, and LDH levels	0.870 (95% CI, 0.801–0.939, *p* < 0.001)
Cheng Q et al. (2021) [114]	China	MP	65/141	WBC count, ALB level, consolidation range exceeding 2/3 of lung lobes, timing of macrolides, glucocorticoids or fiber bronchoscopy and plastic bronchitis	0.899 (95% CI, 0.848–0.950)

MP, Mycoplasma pneumoniae; HAdV, Human adenovirus; PIBO, Post-infectious bronchiolitis obliterans; PICU, Pediatric intensive care unit; LDH, Lactate dehydrogenase; N, Neutrophil; ALB, Albumin; IMV, Invasive mechanical ventilation; AUC, Area under the curve; CI, Confidence interval; NA, not applicable.

## 7. Conclusions

This review synthesizes current knowledge on the pathogenesis of pediatric PIBO with a specific focus on HAdV and MP. We propose a comprehensive integrative model in which acute immune activation progresses to persistent epithelial injury, dysregulated repair, fibroblast activation, and irreversible bronchiolar narrowing. By systematically comparing pathogen-specific responses, we demonstrate that HAdV and MP converge on the MyD88/NF-κB/MAPK pathways and neutrophil-driven inflammation, but diverge in their initial host engagement (CAR/integrins vs. TLR2/6 and CARDS toxin) and inflammasome activation (TLR9-related vs. NLRP3-related).

Markers such as IL-8, neutrophils, and TGF-β have been consistently reported as elevated across multiple cohorts and studies, leading to their recognition as core drivers of the disease. In contrast, chemokines including IP-10, MIG, and MCP-1 appear to be bystanders that lack consistent replication across different pathogens and geographic regions [31]. Recognition of these core drivers is essential for the development of targeted immunomodulatory interventions.

Currently, there is no clearly defined or globally accepted treatment protocol for PIBO, and management typically combines anti-inflammatory and supportive care. Corticosteroids (systemic or inhaled) remain the most commonly used anti-inflammatory agents, but evidence from controlled trials is limited, and their long-term benefit remains inconclusive [5,14,15]. A recent study reviewing early pulse corticosteroid therapy in children with PIBO reported fewer wheezing exacerbations, reduced hospitalizations, and improved oxygen saturation [95]. Macrolides such as azithromycin are often incorporated into maintenance therapy via the FAM (fluticasone, azithromycin, montelukast) regimen to attenuate neutrophilic inflammation by inhibiting chemotaxis and downregulating IL-8 production [5,14,15].

Current knowledge gaps include the lack of single-cell resolution studies and the absence of animal models for pathogen co-infection. Future research priorities should involve validating miRNAs, which hold promise as non-invasive biomarkers and may form the basis for a new class of therapeutic agents in PIBO. Furthermore, the TGF-β/SMAD pathway stands as a key target for future treatments. Drawing insights from anti-fibrotic strategies used in BOS may provide a roadmap for preventing abnormal airway remodeling in PIBO [48]. Well-designed trials are needed to establish the safety and efficacy of new preventive and therapeutic strategies. Prioritizing the early identification of at-risk individuals and standardizing treatment through high-quality clinical trials will be essential to mitigating the global health burden of this debilitating disease.

## Data Availability

Data sharing is not applicable to this article as no datasets were generated or analyzed during the current study.

## Abbreviations

The following abbreviations are used in this manuscript:

BO	Bronchiolitis obliterans
PIBO	Post-infectious bronchiolitis obliterans
HAdV	Human adenovirus
CAR	Coxsackievirus and adenovirus receptor
RGD	Arginine-Glycine-Aspartic acid
TLR	Toll-like receptor
HMGB1	High mobility group box-1
RAGE	Receptor for advanced glycation end-products
MyD88	Myeloid differentiation factor 88
MAPK	Mitogen-activated protein kinase
IL	Interleukin
TNF-α	Tumor necrosis factor-α
NF-κB	Nuclear factor-kappa B
IP-10	Inducible protein-10
MIP-1β	Macrophage inflammatory protein 1β
MIG	Monokine induced by gamma interferon
MIP-1α	Macrophage inflammatory protein-1α
MCP-1	Monocyte chemoattractant protein-1
IFN-α	Interferon-α
MP	Mycoplasma pneumoniae
CAP	Community-acquired pneumonia
CARDS	Community-Acquired Respiratory Distress Syndrome
SP-A	Surfactant protein A
Th2	T-helper 2 cells
MPP	MP pneumonia
LRR	Leucine-rich repeat
AP-1	Activator protein-1
ROS	Reactive oxygen species
MMPs	Matrix metalloproteinases
MPO	Myeloperoxidase
ANCA	Antineutrophil cytoplasmic antibodies
IL-18R	IL-18 receptor
IL-18BP	IL-18 binding protein
BOS	Bronchiolitis obliterans syndrome
RMPP	Refractory mycoplasma pneumoniae
TIMPs	Tissue inhibitors of metalloproteinases
NETs	Neutrophil extracellular traps
SAECs	Small airway epithelial cells
EMT	Epithelial–mesenchymal transition
LDH	Lactate dehydrogenase
MBL2	Mannose-Binding Lectin 2
ECM	Extracellular matrix
TGF-β	Transforming growth factor-β
PDGF	Platelet-derived growth factor
IGF-1	Insulin-like growth factor-1
SPMs	Specialized pro-resolving mediators
Tregs	Regulatory T cells
VEGF	Vascular endothelial growth factor
α-SMA	α-smooth muscle actin
miRNAs	MicroRNAs
